# From Edmonton to Lantidra and beyond: immunoengineering islet transplantation to cure type 1 diabetes

**DOI:** 10.3389/frtra.2025.1514956

**Published:** 2025-03-20

**Authors:** El Hadji Arona Mbaye, Evan A. Scott, Jacqueline A. Burke

**Affiliations:** ^1^ Department of Biomedical Engineering, Northwestern University, Evanston, IL, United States; ^2^ Department of Biomedical Engineering, NanoSTAR Institute, University of Virginia School of Medicine, Charlottesville, VA, United States; ^3^ SNC Therapeutics Inc., Evanston, IL, United States

**Keywords:** type 1 diabetes (T1D), islet transplantation, β-cell replacement therapy, biomaterials, immunomodulation

## Abstract

Type 1 diabetes (T1D) is characterized by the autoimmune destruction of insulin-producing β cells within pancreatic islets, the specialized endocrine cell clusters of the pancreas. Islet transplantation has emerged as a β cell replacement therapy, involving the infusion of cadaveric islets into a patient's liver through the portal vein. This procedure offers individuals with T1D the potential to restore glucose control, reducing or even eliminating the need for exogenous insulin therapy. However, it does not address the underlying autoimmune condition responsible for T1D. The need for systemic immunosuppression remains the primary barrier to making islet transplantation a more widespread therapy for patients with T1D. Here, we review recent progress in addressing the key limitations of islet transplantation as a viable treatment for T1D. Concerns over systemic immunosuppression arise from its potential to cause severe side effects, including opportunistic infections, malignancies, and toxicity to transplanted islets. Recognizing the risks, the Edmonton protocol (2000) marked a shift away from glucocorticoids to prevent β cell damage specifically. This transition led to the development of combination immunosuppressive therapies and the emergence of less toxic immunosuppressive and anti-inflammatory drugs. More recent advances in islet transplantation derive from islet encapsulation devices, biomaterial platforms releasing immunomodulatory compounds or surface-modified with immune regulating ligands, islet engineering and co-transplantation with accessory cells. While most of the highlighted studies in this review remain at the preclinical stage using mouse and non-human primate models, they hold significant potential for clinical translation if a transdisciplinary research approach is prioritized.

## 1 Introduction

Type I diabetes (T1D) is an autoimmune disease that destroys pancreatic β cells, resulting in an inability to produce insulin. In the United States, approximately 2.07 million people are affected by T1D (1). While exogenous insulin remains the standard treatment, issues including patient adherence, severe hypoglycemic episodes, and complications such as blindness and amputations (2) have underscored the need for alternative therapies (3). Islet transplantation emerged as an alternative surgical procedure involving the isolation and purification of islet cells from a donor pancreas, followed by their infusion into the recipient's portal vein. Islet transplantation is an attractive therapy for T1D as it is a long-term solution that can restore physiological glucose control and potentially eliminate T1D complications (4). However, between 2000 and 2020, only 642 islet transplantations were performed in 255 patients in the United States (5) given complications linked with long-term immunosuppression (6) and regulatory hurdles which represent major roadblocks to its widespread adoption in the clinic. Long-term immunosuppression is required to prevent allograft rejection, despite the increased susceptibility to opportunistic infections, malignancies (7) and toxicity to the transplanted islets (8). As the risks of chronic immunosuppression may be more nefarious than those of T1D, islet transplantation has been limited to patients with severe hypoglycemic unawareness. However, islet transplantation has been shown to significantly improve health-related quality of life, with sustained reductions in diabetes-related distress and fear of hypoglycemia for up to 6 years post-transplant (9, 10). These long-term improvements in patient well-being, along with better glycemic control and protection from severe hypoglycemic events, highlight the potential of islet transplantation and has motivated significant research efforts towards the development of transplantation immunotherapies that can induce antigen-specific tolerance for the islet graft without compromising the host's functional protective immunity. In transplantation immunotherapies, antigen-specific tolerance, often regarded as the “holy grail,” differs from the more clinically relevant operational tolerance, which refers to graft acceptance without immunosuppressive drugs but lacks demonstration of true immunological tolerance via third-party donor antigen challenges (11). In this review, we first provide an overview of the current state of therapeutics for enabling islet transplantation, beginning with the establishment of the Edmonton Protocol in 2000, a groundbreaking steroid-free immunosuppressive regimen with a combination of induction and maintenance therapies. We trace the progress up to the Food and Drug Administration (FDA) approval of Lantidra (donislecel-jujn) in 2023, the first FDA-approved allogeneic cellular therapy made from donor pancreatic islet cells for the treatment of T1D, while highlighting the remaining challenges that must still be addressed for widespread clinical adoption. We then discuss recent advances in the development of biomaterial approaches from porous scaffolds to nanoparticles delivering immunomodulatory agents or modified to present ligands capable of dampening immune response towards the islets. Additionally, recent endeavors in islet cell modification and co-transplantation strategies with accessory immune regulating cells are underlined. Finally, we summarize the key advantages of the highlighted approaches (Figure 2), the challenges preventing clinical translation, and advocate for a transdisciplinary research approach that could accelerate the development of transplant immunotherapies without the major side effects of systemic immunosuppression and lead to long-term insulin independence for islet transplant recipients.

## 2 Current therapeutic approaches and their shortcomings

In 2000, Shapiro et al. published the pioneering Edmonton protocol and established islet transplantation as a potential alternative treatment for type I diabetes with all seven patients attaining sustained insulin independence following the procedure (12). Developed at the University of Alberta in Edmonton, Canada, the Edmonton Protocol was the first glucocorticoid-free immunosuppressive regimen for islet transplantation. This steroid-free approach significantly improved the quality of life for patients and established islet transplantation as a viable clinical option. The removal of steroids, which were previously used in immunosuppressive protocols, was crucial as their side effects—such as beta cell damage and insulin resistance—were counterproductive to the success of islet transplants. The protocol, consisting of sirolimus, low-dose tacrolimus, and daclizumab, was assessed in a clinical study including seven patients each receiving islets from at least two donor pancreases. Sirolimus (i.e., rapamycin) works by inhibiting the mammalian target of rapamycin (mTOR), an essential protein kinase involved in regulating cell growth, metabolism and survival. This inhibition prevents T-cell proliferation by blocking the T-cell response to IL-2. Meanwhile, tacrolimus binds to FKBP-12, inhibiting calcineurin, which reduces IL-2 production and thereby prevents T-cell activation. Daclizumab, an anti-CD25 monoclonal antibody, blocks the IL-2 receptor on T cells, inhibiting activation and proliferation. This combination therapy was chosen to avoid usage of glucocorticoids, as this drug class has been linked with beta cell damage, reduced C-peptide levels at high dosage and increased insulin resistance (13). However, in 2018, pharmaceutical companies AbbVie and Biogen withdrew daclizumab from the global market, as a result of reports of serious inflammatory brain disorders (14). Sirolimus, tacrolimus, and daclizumab synergistically prevent the activation of T cells, the production of IL-2 and its downstream effects on clonal expansion of lymphocytes. Shapiro and colleagues reported that patients in the study remained free from cytomegalovirus infection, hyperlipidemia (often associated with sirolimus), and other significant side effects typically linked to chronic use of this immunosuppressive regimen (12).

Shapiro's follow-up study in 2006 with a larger number of participants highlighted a different story. The study reported multiple immunosuppression-related adverse events such as neutropenia, pneumonia, gastrointestinal conditions. Additionally, 25% of patients required a change of therapy, which accelerated their withdrawal from the study (15). These results underscore the urgent need for less toxic immunotherapies, as prolonged exposure to sirolimus and tacrolimus will lead to significant accumulation within erythrocytes [94.5% for sirolimus (16) and 85%–95% for tacrolimus (17)] and eventual deposition in off-target organs. As such, these drugs can cause islet toxicity in the portal vein (18), the site of islet infusion. Hence, significant efforts have focused on developing immunosuppressive drugs that specifically target immune cell pathways, aiming to minimize toxicity to islets and other organs following transplantation. Two critical pathways under investigation are the CD28-B7 pathway and the CD40-CD40l pathway. Belatacept, a high-affinity variant of cytotoxic T lymphocyte-associated antigen-4 immunoglobulin (CTLA4-Ig), received FDA approval in June 2011 for use in kidney transplantation (19). Belatacept binds to CD80 (B7-1) and CD86 (B7-2) and prevents stimulation of CD28 leading to T cell anergy and apoptosis (20). Belatacept does not deplete T cells, is shown to upregulate Tregs in graft biopsies of kidney transplant recipients (21) and protects patients against renal, cardiovascular and metabolic adverse events encountered with calcineurin inhibitors (CNIs) (i.e., tacrolimus) (18). In a 2010 study by Posselt et al., four out five belatacept-treated diabetic patients became insulin independent after a single transplant while one resumed insulin injections after 305 days but later became independent after a second transplant (22). As these authors reported no serious adverse events (22), belatacept emerges as a promising alternative immunotherapy to enhance graft function and longevity in islet transplantation. However, larger study cohorts and extended follow-up studies are necessary for widespread clinical adoption. In the pursuit of less toxic immunosuppressive therapies, anti-CD40l antibodies have gained considerable research interest. Recent advances leveraging the CD40-CD40l blockade are discussed in greater detail in this review (23). While thromboembolic complications observed in preclinical and clinical settings hampered initial progress, anti-CD40l antibodies such as Tegoprubart (AT-1501) were engineered to reduce binding to Fcγ receptors on platelets while preserving affinity to CD40l (24). Recent work by Kenyon and colleagues demonstrated that, although monotherapy with AT-1501 alone was insufficient to achieve long-term graft survival in nonhuman primates (NHPs), combining AT-1501 with thymoglobulin induction led to graft survival for at least 55 days in three out of four NHPs. Compared to those under conventional immunosuppression, animals treated with AT-1501 exhibited higher fasting and meal-stimulated corrected C-peptide levels, gained weight, and avoided severe cytomegalovirus reactivation (24). Despite limitations in the study's statistical power, AT-1501 shows reduced toxicity compared to conventional immunosuppressive regimens (24) and is likely to progress further into clinical testing for islet transplantation. Nonetheless, several key gaps will still need to be addressed before anti-CD40l drugs like AT-1501 can enter clinical use, including demonstration of long-term efficacy and safety, optimization of dosing and combination strategies.

**Figure 1:**
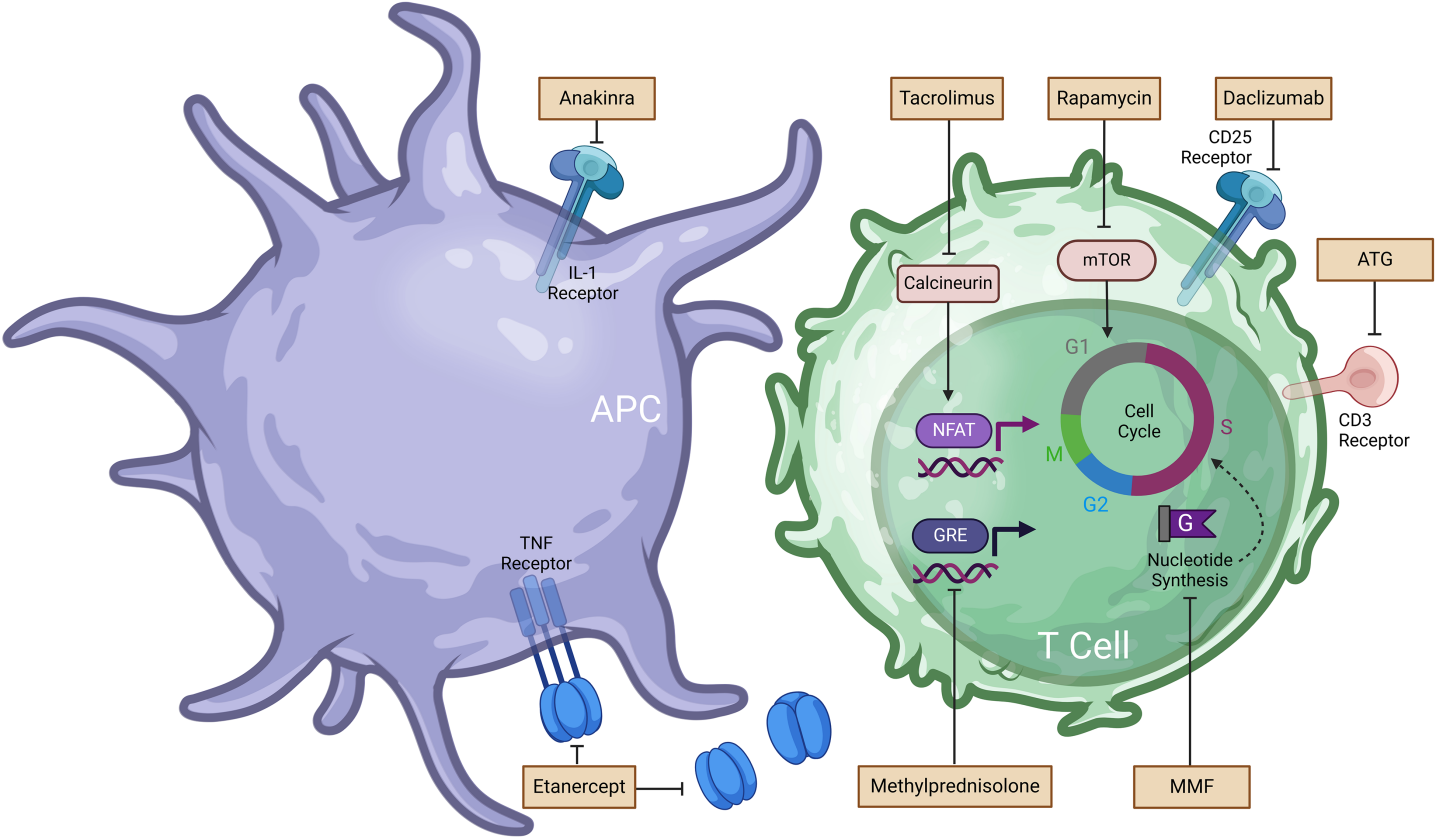
Immunosuppressive and anti-inflammatory drugs used in islet transplantation and associated mechanism of action (MoA). Further details regarding MoA are summarized in Table 1. Anakinra prevents the binding of interleukin-1 (IL-1) to the IL-1 receptor. Etanercept blocks the action of tumor necrosis factor (TNF). Tacrolimus inhibits calcineurin activity and downstream nuclear factor of activated T cells (NFAT), resulting inhibition of T cell activation. Rapamycin binds to the protein kinase mammalian target of rapamycin (mTOR) and arrest T cell activation and proliferation. Daclizumab binds to the CD25 receptor on T cells and impedes IL-2 mediated T cell activation. Methylprednisolone hinders the transcription of inflammatory genes. Mycophenolate mofetil (MMF) prevents *de novo* guanine nucleotides synthesis required for T cell and B cell proliferation.

More than 20 years after Shapiro's pioneering work, Marfil-Garza et al. published Shapiro's 20 year-follow up results. Marfil-Garza et al. study of 255 islet recipients which demonstrated a median graft survival time of 5.9 years and the safety of islet transplantation procedure despite the side effects of chronic immunosuppression (25). The use of anti-inflammatory drugs anakinra [anti-interleukin-1 (IL-1)] and etanercept [anti-tumor necrosis factor (TNF)] aimed at preventing rapid islet loss following infusion, was the best predictor for prolonged graft survival (8). These anti-inflammatory drugs were developed to address another major obstacle to the widespread implementation of islet transplantation which is the need for 2 or more donor pancreases in order to achieve insulin independence (12). Poor engraftment of transplanted islets has been attributed to multiple factors including islet quality and transplant site, however, the instant-blood-mediated inflammatory response (IBMIR) has been deemed a major contributor to the necessity of multiple donors for a single recipient (26). IBMIR occurs during the contact of islets with the blood in the portal vein and leads to the rapid loss of over 60% of transplanted islets within hours to days post-transplantation (27). Coagulation, complement activation, and the release of pro-inflammatory cytokines such as tumor necrosis factor-alpha (TNF-α) and interleukin-1 beta (IL-1β) by transplanted islets culminate in the infiltration of the recipient's polymorphonuclear cells (i.e., neutrophils) (26, 28). The resulting inflammatory response and coagulation due to platelet activation leads to thrombosis throughout the liver's vasculature. As such, islets do not receive an adequate supply of nutrients and oxygen, causing necrosis stemming from the islet core and resulting in apoptosis. Islet death further increases the requirement for multiple infusions for transplant recipients to achieve normoglycemia (29). To address this inflammatory cascade, anti-inflammatory agents such as anakinra and etanercept have been thoroughly investigated. Etanercept is a fusion protein that inhibits TNF-α and its interaction with cell-surface TNF-receptors. Anakinra is an IL-1 receptor antagonist that prevents β-cell destruction and minimizes IBMIR (8). In their 2005 study, Hering et al. demonstrated the possibility of single donor islet transplantation with five out of eight patients showing insulin independence for more than 1 year. The induction immunosuppressive regimen was comprised of rabbit antithymocyte (rATG), methylprednisolone, daclizumab, and etanercept while the maintenance regimen included sirolimus and low-dose tacrolimus until it was later replaced with mycophenolate mofetil, which inhibit T and B cell proliferation by preventing *de novo* synthesis of guanine nucleotides (30, 31). Hering and al. argued that the low islet dose required for normoglycemia was solely due to the addition of etanercept, given that it represented the only modification to the protocol compared to their previous trial (32). Hering and colleagues' results with a single islet donor were further substantiated by Matsumoto et al. In a study of 3 patients, Matsumoto and colleagues showed that a sirolimus-free protocol comprised of rATG, anakinra, etanercept, and mycophenolate mofetil only required a single islet infusion for long-term insulin independence (>1 year) (33). To evaluate the synergistic effects of an anakinra and etanercept model, McCall et al. transplanted human islets in an immunodeficient mouse model and found that combination therapy significantly increased islet engraftment to 87.5% compared with 45.5% with etanercept alone or 53.9% with anakinra alone (34). The synergy of IL-1 and TNF-α blockade could be explained by TNF-α potentiating effect on IL-1, which can lead to β-cell death via augmentation of the mitogen-activated protein kinase signaling pathway (35). Chronic administration of etanercept and anakinra combination therapy is associated with higher incidence of severe infection (7%) in rheumatoid arthritis patients when compared to the monotherapy (1.8%) (36, 37). While these drugs are usually administered over 1 week in the post-transplant period, recipients are also subjected to extensive T cell depletion via the chronic use of maintenance immunosuppressants (34); hence the risk of infection following treatment with etanercept and anakinra does merit further investigation to establish all potential risks. Notable side effects for conventional immunosuppressive and anti-inflammatory drugs used in islet transplantation are outlined in Figure 1 and Table 1, along with information of the mechanism of action. While anti-inflammatory agents have become integrated into the routine peritransplant management of islet transplant recipients, the randomized controlled clinical trials needed for clinical translation are faced with scarce donor availability (34). To overcome this challenge, attention has been shifted towards novel cell sources and cell-based therapies.

**Table 1:** Immunosuppressive and anti-inflammatory drugs used in islet transplantation and associated mechanism of action (MoA).

Brand name/ pharma name	Route of administration/formulation	Class	MoA	Notable side effects	Reference
Rapamune/Rapamycin (sirolimus)	Oral tablet and oral solution	mTOR inhibitor	Arrest T cell activation and proliferation by inhibiting progression from G1 to S phase	Latent viral infections Nephrotoxicity Hyperlipidemia	(38)
Prograf/Tacrolimus	Oral capsules and intravenous use	Calcineurin inhibitor	Inhibits IL-2 receptor expression and nitric oxide release on T cells	Increased risk of cancer Increased risk of infection	(39)
Zinbryta/Daclizumab (anti-CD25)	Subcutaneous use	IL-2 receptor blocking antibody	Binds to CD25 and prevents IL-2 mediated T cell activation	Autoimmune liver problems Inflammatory brain disorders	(40)
Thymoglobulin/Antithymocyte globulin	Intravenous use	Immunoglobulin	T cell clearance and modulation of T cell activation	Anaphylactic reaction Low platelet and white blood cell count	(41)
Medrol/Methylprednisolone	Intravenous and Intramuscular use	Glucocorticoid	Binds to the intracellular glucocorticoid receptor and blocks the transcription of inflammatory genes in immune cells	Gastritis Gastrointestinal bleeding	(42)
CellCept/Mycophenolate mofetil	Oral tablets, oral suspension and intravenous use	IMPDH inhibitor	Prevents guanine nucleotides synthesis critical for T and B cell proliferation	Low blood cell count Stomach problems	(43)
Kineret/Anakinra	Subcutaneous use	IL-1 receptor antagonist	Binds to the IL-1 receptor on macrophages and lymphocytes and suppresses inflammation	Worsening of rheumatoid arthritis Upper respiratory tract infection	(44)
Enbrel/Etanercept	Subcutaneous use	TNF blocker	Prevents TNF cytokines from binding its receptor on macrophages and lymphocytes	Infection risk Injection site reaction	(45)

Deriving islets from embryonic stem cells (ESCs) is a feat that has been explored since the beginning of this century. Foundational research includes the generation of pancreatic endoderm cells (PECs)—multipotent cells derived from embryonic stem cells (ESCs) during embryonic development, as demonstrated by D'Amour et al. (46). Additionally, Kroon et al. reported the successful transplantation of insulin secreting cells from human ESCs capable of maintaining normoglycemia in a murine model (47). These results set the stage for companies such as ViaCyte and Vertex to explore the translatability of using alternative cell sources for combating organ shortage in islet transplantation. ViaCyte launched its initial clinical trial in 2014 with a combination product known as VC-01. VC-01 consists of PECs encapsulated within a fully protective polytetrafluoroethylene (PTFE) membrane, designed to safeguard the cells against the host's immune response (48). While the need for chronic immunosuppression was removed in this context, the trial was discontinued due to “insufficient functional product engraftment” which suggests that the membrane did not allow for adequate supply of nutrients and oxygen to maintain cell viability (48, 49). Later in 2021, ViaCyte modified VC-01 to develop the VC-02 device, another functional cure pipeline to be semi permeable, facilitating vascular growth to support oxygenation and metabolic processes (3). Six of the seventeen patients implanted with VC-02 showed detectable C-peptide levels (>0.1 ng/ml) as early as 6 months post-transplant and significant insulin secretion from the transplanted cells (3). To build upon this work, Vertex acquired ViaCyte in 2022 with the goal of accelerating its curative treatment. However, the semi-permeable nature of the VC-02 reestablished the need for immunosuppression which was linked to a significant incidence of side effects (33.7%) (3). Furthermore, the differentiation pathway of PECs was uncontrolled and this hurdle may explain the insufficient insulin levels as well the high abundance of glucagon-expressing cells (49). To avoid variable differentiation of PECs resulting in undesirable cell populations, Vertex Pharmaceuticals opted to transplant fully differentiated ESCs-derived islet cells (VX-880) via intraportal infusion in a single patient at half the required dose and showed increases in fasting and stimulated C-peptide (small protein fragment produced alongside insulin) as well improvements in insulin independence (50). These results were further substantiated in 2023 when two patients followed during the first year of the study became insulin-independent, showed glycated hemoglobin levels (HbA1c) <7% and maintained 95% time-in-range based on continuous glucose monitoring (51). While Vertex's VX-880 was well tolerated, the therapy was only trialed in a limited number of patients. Furthermore, there was still a need for chronic immunosuppression (49). To eliminate the need for chronic immunosuppression, Vertex launched VX-264 (52) which uses the same ESCs derived islets cells as VX-880 with the addition of a protective channel array device to shield the islets from the recipient's immune system, while allowing the diffusion of oxygen and nutrients. Initial dosing began in March 2023 and the phase 1/2 trial is still ongoing (52). In addition to these islet encapsulation devices undergoing clinical trials, many research efforts have been produced towards the use of physical barriers to protect the islets against the host's immune cells. These efforts have been reviewed in greater detail elsewhere (53).

Like the approach of deriving insulin producing islet cells from ESCs, numerous reports in the literature have shown the successful derivation of β-like cells from pluripotent stem cells (54–56). These cells offer a promising avenue for islet transplantation as they can be generated from a patient's own induced pluripotent stem cells, potentially providing an unlimited source of insulin-producing cells for transplantation (57). The use of the autologous pluripotent stem cells theoretically eliminates the need for long-term immunosuppression to prevent allogeneic rejection. However, the literature remains unclear whether immunosuppression would be still required given differences in differentiation protocols and potential remains of xenogeneic material in the *in vitro* differentiation culture (58). Hence, researchers suggest that even with autologous pluripotent stem cell-derived β-Like cells, life-long immunosuppressive therapy may be necessary to protect against autoimmune rejection due to the hyperactive immune response in patients with T1D (59). This uncertainty highlights the need for further research to determine the optimal approach for immune protection in autologous pluripotent stem cell-derived β-Like cells transplantation and the development of manufacturing processes and release criteria that will be essential for clinical translation (58).

While the work for alternative cells sources for islet transplantation continues, on June 28, 2023 the FDA approved the use of Lantidra as the first allogeneic (deceased donor) pancreatic islet cell therapy for patients with T1D who do not achieve target HbA1c levels (60). As of the writing of this manuscript, Lantidra is exclusively available at the University of Illinois (UI) Health in Chicago (61). Patients eligible to receive Lantidra must be at least 18 years of age, be diagnosed with T1D for more than five years, be insulin dependent and have a body mass index (BMI) lower than 27 (61). Patients must have also experienced severe hypoglycemic episodes in the past three years despite insulin treatment and present no other serious health concerns or active infections (61). Like institutionally isolated islets, Lantidra is given as an infusion via the portal vein. In clinical studies, after receiving Lantidra, 21 of the 30 participants did not require insulin for 1 year or longer. The most adverse side effects were linked to the need for chronic immunosuppression and the hepatic vein perfusion procedure, especially when multiple infusions were needed (62).

The need for chronic immunosuppression and its severe adverse events remains a common drawback for most transplant immunotherapies. Consequently, significant research efforts have focused on developing novel therapies that can establish specific immune tolerance towards transplanted islets while maintaining functional protective immunity (49). Below, we compare the recent preclinical research in immunomodulation via biomaterials-based approaches to islet engineering and cellular co-transplantation strategies, which allow islet cells to protect themselves against the host's immune system.

## 3 Immunomodulation via biomaterials-based approaches

Rapid advances and convergence of expertise in biomaterial sciences and immunology have led to the development of multiple strategies aimed at inducing tolerance to allogeneic islets without the need for systemic immunosuppression. By tuning biomaterial properties such as size, shape and surface chemistry (63), it is possible to create local immune privileged microenvironments or target specific immune cells in the draining lymph nodes. The most commonly investigated polymeric biomaterial is poly(lactic-co-glycolic acid) (PLGA) as it is used in multiple FDA approved cancer therapies (64) and has served as the delivery vehicle for the formulation of multiple tolerance-inducing therapies (65–68). Biomaterials strategies for promoting islet transplantation tolerance typically focus on two approaches: the controlled release of small molecule drugs and proteins, and the conjugation of immunomodulatory ligands on the surface of biomaterials (69, 70). These strategies can be further categorized into local immunomodulation for avoidance of systemic side effects and targeting of antigen-presenting cells in the lymph nodes (66, 71). All of these methods have significantly improved the survival and function of transplanted islets in preclinical models and are summarized in Table 2.

**Table 2:** Examples of biomaterial approaches for improved graft survival in islet transplantation.

Biomaterial/delivery system	Cargo released/modification	Route of administration/islet transplant site	Notable immunomodulatory outcomes	Reference
PLGA micelles	Dexamethasone + CTLA4-Ig	Co-delivery with islets under the kidney capsule	80% of transplant recipients remaining normoglycemic for up to 60 days Decrease in proinflammatory cytokines	(67)
PEG-b-PPS Polymersomes (rPS)	Rapamycin	Subcutaneous administration with intraportal islet transplantation	10/12 mice normoglycemic up to 100 days via downregulation of costimulatory molecules CD80, CD86 and CD40 on APCs and upregulation of CD8+ Tregs in axial/brachial lymph nodes	(71)
PLGA microparticles	Rapamycin and Erythrocyte alloantigen (Ea)	Intra-lymph node delivery with islets transplanted under kidney capsule	Graft survival extended up to 56 days with Treg induction and changes in lymph node microenvironment	(66)
PLGA microspheres	FK506	Co-delivery with islets in the subcutaneous space	60% of recipients remained normoglycemic via inhibition of cytotoxic T cells and macrophages	(68)
Liposomes	Clodronate	Islets delivered within Matrigel and co-delivered subcutaneously with liposomal clodronate	83.33% of mice remained insulin- independent for more than 60 days through macrophage depletion and lower T eff cells infiltration	(72)
PLG scaffold	IL-33	Islets seeded on loaded scaffold and delivered to epididymal fat pad	Median survival time increased to 33 days and upregulation of Foxp3+ T cells and decrease of CD8+ T cells	(73)
Human-derived acellular dermal matrix	CTLA4/Fc	Islets between strips of matrix and placed under the kidney capsule	Median survival time extended to 71 days and induction of Tregs locally and systemically	(74)
Blend of PLGA and PBAE	anti-CD3, anti-CD28 and TGF-β	Co-delivery with islets subcutaneously	Graft failed after a few days but expansion Tregs via costimulation blockade	(75)
PEG microgels	SA-FasL	Co-delivery with islets in the epididymal fat pad and omentum for murine and nonhuman primate models respectively	12/13 mice remain normoglycemic for up to 200 days All four NHPs show glycemic control, sustained C-peptide levels and lower insulin requirement Increase in ratio of Tregs to Teff cells Short, low dose rapamycin course was critical to success	(69, 76)
PLG scaffold	SA-FasL	Co-delivery with islets in the epididymal fat pad	Significant extension of graft survival up to 200 days Short, low dose rapamycin course was critical to success	(77)
PEG-hydrogel	SA-FasL+ IL-2	Co-delivery with islets in the epididymal fat pad	Rapid graft failure but showed high Treg levels IL-2 also expanded Granzyme B + T eff cells	(78)
PEG microgels	SA-PD-L1	Co-delivery with islets in the epididymal fat pad	60% of recipients normoglycemic for >100 days Short, low dose rapamycin course was necessary	(70)
Mesenchymal stem cell membrane-derived vesicle-crosslinked hydrogel (MMV-Gel)	FasL+ PD-L1	Co-delivery with islets under the kidney capsule	66% of recipients were insulin-independent for up to 30 days and short, low dose rapamycin course extended graft survival for up to 100 days	(79)

### 3.1 Immunomodulatory agent-eluting biomaterials

Extensive research has gone into the use of scaffolds, hydrogels, nanoparticles, and microparticles for the delivery of immunomodulatory agents (80–82). Local delivery of these agents to the islet graft is prioritized to establish a local immunosuppressive or immunotolerant milieu and minimize adverse effects associated with systemic administration while achieving higher drug concentrations directly within the transplant microenvironment (80). Nonetheless, localized delivery of anti-rejection drugs necessitates islet transplantation in confined extrahepatic sites (65), such as the subcutaneous space or the anterior chamber of the eye which are still under investigation for clinical use. This approach is crucial to prevent drug metabolism in the liver and minimize systemic redistribution to off-target organs. When systemically exposed at high doses, dexamethasone is a potent glucocorticoid that can inhibit insulin secretion from beta cells (83). However, at low doses, dexamethasone released from macroporous scaffolds can preserve beta cell mass and accelerate islet cell engraftment (84). As such, Pepper and colleagues encapsulated dexamethasone into PLGA micelles and supplemented the therapy with four intraperitoneal injections of CTLA4-Ig fusion protein. The study demonstrated that 80% C57BL/6 mice recipients remained normoglycemic for 60 days following allogeneic islet transplant (67). By co-delivering the islets with the loaded micelles in the portal vein of diabetic mice, the authors found reduced expressions of pro-inflammatory cytokines such as IL-1β and IFN-γ in the graft microenvironment. However, while the combination of dexamethasone-loaded micelles and CTLA4-Ig doubled the allograft survival compared to empty micelles, significance was not achieved (67) suggesting that more potent active agents may be required.

Immunosuppressive drugs have also been combined with biomaterials to further dampen immune activation against the allograft by targeting drug specifically to antigen-presenting cells (APCs) which modulate the activation of T cells and their subsequent role in allograft rejection (85). Rapamycin is a commonly used immunosuppressant in allogeneic islet transplantation studies given that it can inhibit T cells via cycle arrest in the G1 phase (86), but can also lead to the development of a tolerogenic phenotype in dendritic cells (DCs) and the upregulation of regulatory T cells (Tregs) (87). As such, rapamycin was loaded into poly(ethylene glycol)-*block*-poly(propylene sulfide) (PEG-*b*-PPS) bilayer 100 nm vesicles (i.e., polymersomes) and subcutaneously administered into streptozotocin-induced diabetic mice for passive targeting of antigen-presenting cells (71). Burke et al. found that this subcutaneous nanotherapy of rapamycin-loaded polymersomes (rPS) led to 83% of diabetic mice remaining normoglycemic 100 days post-transplant. PEG-*b*-PPS was utilized in this work as it was found to be nontoxic to human islets (88), and rPS enhances the passive targeting and induction of relevant tolerogenic APC populations (71). A short regimen of rPS, six 1 mg/kg administrations over 15 days, resulted in the downregulation of costimulatory molecules CD80, CD86, CD40, and the upregulation of MHC II on APCs in the axial and brachial lymph nodes. Subsequently, CD4+ T cells became anergic and CD8+ Tregs, which are critical for immune homeostasis and tolerance, were upregulated (71). A mixed lymphocyte reaction (MLR) showed that mice treated with rPS demonstrated reduced response towards donor antigens while maintaining a proliferative response towards non-donor T cells (71). This led the authors to conclude that by targeting APCs in the draining lymph nodes, the targeted nanotherapy can alter the mechanism of action of rapamycin from broad immunosuppression to antigen-specific tolerance. This concept was further explored by co-loading rapamycin and the major histocompatibility alloantigen erythrocyte alloantigen (Ea) in PLGA microparticles (MPs) and using intranodal delivery to the inguinal lymph nodes (64). In the fully MHC-mismatched islet transplantation mouse model, Jewell and colleagues found that the Ea/rapamycin MPs significantly extended survival to 56 days post-transplantation (v. 21 days for control) and resulted in structural changes to the lymph nodes microenvironment that are conducive to the induction of memory Tregs induction and reduced differentiation of Th1 and Th17 phenotypes (66). The use of MPs and intranodal delivery enhanced the concentration of immunosuppressive cues in the lymph nodes leading to durable and systemic antigen-specific tolerance (66).

FK506 (i.e., Tacrolimus) is another immunosuppressant explored for islet transplantation given its capacity to inhibit T cell signal transduction and IL-2 transcription required for T cell proliferation (89). In a study by Pathak et al., FK506 was encapsulated into 5 μm PLGA microspheres and subcutaneously co-delivered alongside islets encapsulated within Matrigel, a hydrogel composed of extracellular matrix components derived from murine sarcoma cells. Given the paucity of blood vessels in the subcutaneous space, the presence of Matrigel or other viability matrices are critical for vascularization and fulfillment of the oxygen and nutrient requirements of the islet cells (90). Nonetheless, necrosis of islets can occur during vasculogenesis, which has led others to opt for a preconditioning approach for islet transplantation in the subcutaneous space (91). This single synchronous delivery system led to normoglycemia in 60% of mice for up to 30 days via the strong inhibition of cytotoxic T cell and macrophage proliferation (68). Local immunomodulation by FK506 loaded microspheres was necessary as administration of the islets on one flank and the loaded microspheres to another flank of the mice failed to reach significance compared to the control group (68). In another attempt to prevent early islet damage in the portal vein, clodronate, a macrophage-depleting agent was investigated given that macrophages play a significant role in IBMIR in the liver (92). Using Matrigel for islet delivery, researchers formulated clodronate in a liposomal formulation and found that co-delivery with islets resulted in 83.33% of mice recipients remaining normoglycemic for more than 60 days (91). Additionally, the liposomal clodronate resulted in significantly lower infiltration of CD4+, CD8+ and CD11b+ cells into the Matrigel and reduced concentration of TNF-ɑ and IL-1β in the islet microenvironment (72). While clodronate can cause adverse effects such as abdominal pain and osteonecrosis (72), clodronate released from dead macrophages cannot penetrate other cells and authors believe that killing of macrophages in the early stages of the immune reaction against islet cells can promote adaptation of the immune system and induction of tolerance (72). The clinical translation of these approaches may be limited by the use of Matrigel, which is associated with safety concerns due to its mouse tumor-derived origin and reported batch-to-batch variability (93).

Aside from immunosuppressive drugs, cytokines, and fusion proteins have also been combined with biomaterials in the pursuit of superior immunotherapies for islet transplantation. IL-33 is a cytokine from the IL-1 family that has both pro- and anti-inflammatory properties (94). However, in the context of allogeneic transplantation, IL-33 has prevented rejection in murine cardiac transplant models via upregulation of T helper type 2 (Th2) and Treg responses (95, 96). Thus, Liu et al. formulated a 75:25 poly(lactide-co-glycolide) (PLG) scaffold loaded with IL-33 and implanted in the epididymal fat pad along with allogeneic islets. IL-33 release from the scaffold led to a significant increase of Foxp3+ (Treg marker) cells expressing the ST2 receptor (Interleukin 1 Receptor-Like 1) and a notable decrease of CD8+ T cells in the allograft environment (73). Effects of the cytokine were local as such changes were not observed in the spleen. Additionally, IL-33 induced a Th2 cytokine response shifting T cell polarization from Th1 to Th2 and leading to a significant increase in median survival time from 14 to 33 days for IL-33 loaded scaffolds compared to control scaffolds (73). In another study, nanogram quantities of murine CTLA4/Fcγ2α heavy chain chimeric fusion protein (CTLA4/Fc) were bioprinted onto 5 × 5 mm pieces of human-derived acellular dermal matrix (ADM). Islets resided between the pieces of ADM such that biopatterned CTLA4/Fc could be in direct contact with the islets during the transplant procedure (74). CTLA4-Ig lead to costimulation blockade by binding to CD28 and impeding T cell activation (97). As such, Solari and colleagues found that local delivery of CTLA4/Fc led to a median survival time of 71 days in the MHC-mismatched DBA2 to C57BL/6 mice islet transplantation model compared to control mice with a median survival time of 15.5 days (74). Expression of pro-inflammatory cytokines such as IL-6 and IFN-γ decreased in the islet microenvironment along with a marked upregulation of CD4+ Foxp3+ Tregs in the islet allografts, peripheral blood and draining lymph nodes compared to the non-treated control (74). In an attempt to drive T cell differentiation towards the Treg phenotype, Neshat and al. fabricated 0.2 μm artificial APCs composed of a blend between PLGA and poly(beta-amino ester) (PBAE), coated with anti-CD3 and anti-CD28 and loaded with TGF-β. While subcutaneous co-injection of the artificial APCs with allogeneic islets stimulated the expansion of Tregs, high levels of CD8+ T cells and NK cells infiltrated the graft preventing the restoration of normoglycemia (75).

### 3.2 Immunomodulatory ligands on biomaterials surface

Surface modification of biomaterials with ligands capable of binding immune cells and eliciting changes in immune cell population in the islet microenvironment present an attractive route for inducing tolerance of allogeneic islets. Fas ligand (FasL) and programmed death ligand 1 (PD-L1) have been the most explored ligands for surface modifications given their previously reported role in halting or reversing T1D pathogenesis in non-obese diabetic (NOD) mouse models (98, 99). FasL binds to the Fas receptor which is upregulated on T cells following activation and plays a significant function in activation-induced cell death and tolerance to self-antigens (100). PD-1 plays a critical role in T cell exhaustion and its binding to PD-L1 inhibits T cell activation. As such the PD1/PDL-1 pathway has been extensively studied for cancer applications (101), but its potential role in allogeneic transplantation models (102) has made it a target for the development of novel immunotherapies. Given that systemic administration of Fas and PD1 ligands have been associated with immune-related toxicity (101), biomaterial approaches have largely focused on hydrogels or scaffolds co-delivered with the islets for local immunomodulation. Biotinylated poly(ethylene glycol) (PEG) microgels capable of capturing streptavidin/FasL chimeric protein with high affinity extended islet allograft survival with twelve out of thirteen mice remaining normoglycemic up to 200 days post-transplant (69). A short course of rapamycin at a much lower dose than required for graft survival was required for optimal efficacy. Graft survival was dependent on an increase in the ratio of Treg to CD4+ and CD8+ T effector (Teff) cells in the graft and draining lymph nodes as Treg depletion exacerbated islet rejection times (68). Given the promise demonstrated by PEG microgels, Garcia and colleagues tested the FasL-conjugated PEG microgels in nonhuman primates (NHPs). Following allogeneic islet transplantation, rapid glycemic control was achieved in all four NHPs and lasted for up to 6 months (76). These results were significantly different from the microgels lacking FasL and graft survival was associated with upregulation of Tregs at the graft site while T cell populations remained unchanged systemically. While rapamycin was required for 2 weeks in the mouse model, the rapamycin regimen was extended to 3 months for NHPs and FasL microgels recipients still required exogenous insulin, albeit the insulin requirement was only 10%–20% of the pretransplant dose for maintenance of normoglycemia (76). Similar to their approach with PEG microgels, the same research group fabricated PLG scaffolds, designed to enhance nutrient diffusion and promote rapid islet revascularization (77). The co-delivery of the islets with the scaffold displaying streptavidin/FasL along with a short course of rapamycin led to a comparable outcome as the PEG microgels in a murine allogeneic islet transplantation model. Furthermore, a comparison of the surface-modified PLG scaffolds with islets engineered to display FasL showed no significant difference in graft survival compared to unmodified islets (77), avoiding possible complications that would arise from genetic modification of the islets as an off–the-shell product.

To circumvent the need for the short course of low-dose rapamycin, Garcia and colleagues combined the FasL conjugated microgels with a protease degradable IL-2 loaded PEG hydrogel. IL-2 was used for its capacity to stimulate Treg expansion, given that unlike CD8+ T cells and NK cells, Tregs are more sensitive to IL-2 due to the expression of the high-affinity ɑ-chain of the IL-2 receptor (103). Furthermore, IL-2 enhances FasL-mediated killing of mature alloreactive T cells (104) and thus potentially usher to long-term protection of the islet graft. While the combination therapy of FasL PEG microgels and IL-2-releasing hydrogels significantly upregulated Tregs at the graft site in a fully mismatched islet transplantation model, the combination therapy did not lead to any changes in the levels of CD4+ and CD8+ T cells (78). Surprisingly, the FasL microgels in addition to the IL-2 hydrogel upregulated the levels of CD8+ T cells expressing granzyme B. Given the rapid failure of the graft, the authors concluded that the long half-life of IL-2 (5.15 days) (78) and its potential to stimulate Teff cells (72), should be carefully considered. These factors will be critical for the development of successful IL-2-based therapies for islet transplantation.

PD-L1 was also conjugated onto PEG microgels, and co-delivery with allogeneic islets in the epididymal fat pad resulting in 60% of recipients maintaining normoglycemia for over 100 days (70). Although a brief course of rapamycin helped extend graft survival for the PD-L1-decorated microgels, the use of rapamycin alone led to graft rejection in more than 85% of recipients. Authors showed that PD-L1 presenting microgels create a tolerogenic environment via upregulation of CD4+ Tregs and while they attempted to elucidate possible changes in the myeloid populations, those responses were less pronounced (70). Although the results are promising, significant optimization remains necessary, as the ligand-conjugated microgels without the short course of rapamycin achieved only 22% graft survival beyond 50 days. The generation of Tregs, whether via recruitment or *de novo* generation is yet to be understood. Finally, Wang et al. sought to combine the effects of FasL and PD-L1 into a single therapy via the engineering of membrane-derived vesicles (MMVs) crosslinked into a hyaluronic acid hydrogel. MMVs are obtained through membrane extraction and extrusion of mesenchymal stem cells which express molecules such as FasL and PD-L1 on their surface and have been extensively explored for autoimmune disorders given their potent immunomodulatory properties (105). The expression of these ligands can be modulated by nitric oxide (NO) and inflammatory cytokines such as IFN-γ and TNF-α (105). The combination of FasL and PD-L1 aimed to induce apoptosis of Teff cells via binding of FasL to Fas receptor and upregulate Tregs via the suppression activity of the PD1/PD-L1 pathway on Teff cells (106). As a result, 66% of allogeneic islet recipients remained normoglycemic for up to 30 days following treatment with the MMV-gel and when a short course of rapamycin was added to the MMV-gel treatment, graft survival was observed for up 100 days for 60% of transplant recipients (79). As such, this study highlights that while graft survival was dependent on the upregulation of Tregs in the islet microenvironment, long-term maintenance of Tregs via rapamycin treatment was also critical for significant allograft survival.

Biomaterials-based approaches for islet transplantation provide the advantage of local or targeted delivery of small molecules or biologics as compared to more toxic systemic immunosuppressive therapies. Nevertheless, successful clinical translation will require addressing multiple limitations such as the depletion of the delivered active agent over time and the need for a stronger grasp of the cellular microenvironment of the diverse extrahepatic sites currently being tested in animal models and clinical trials, in addition to the clinical standard intraportal site.

## 4 Islet engineering/co-transplantation strategies

Emerging trends in islet research include cellular therapies involving genetic engineering of islets or co-transplantation of immunomodulatory cells capable of supporting the biological functions of the transplanted islets and suppressing or delaying the immune reactions against the graft. Table 3 outlines recent advances made in islet modifications and cellular co-transplantation strategies for islet transplantation.

**Table 3:** Summary of recent efforts in islet modification and co-transplantation strategies for islet transplantation.

Modification/Immunomodulatory cell	Islet transplant site	Notable immunomodulatory outcomes	Reference
Biotinylated islets + SA-FasL	Kidney capsule	100% graft survival with short course of rapamycin High intragraft levels of Tregs and no chemotactic factor for neutrophils	(107)
Biotinylated islets + SA-PD-L1	Kidney capsule	90% graft survival with 15 day- long, low dose rapamycin High levels of Tregs, TGF-β and IL-10	(108)
Islets + Engineered mesenchymal stromal (eMSCs)	Kidney capsule	Median survival time of 40 days High levels of exhaustion markers and CD4+ Tregs at graft site	(106)
Islets + Sertoli cells	Liver (intraportal)	86% of recipients normoglycemic up to 100 days High levels of insulin positive cells 120 days post-transplant	(109)
Islets + Tregs	Liver (intraportal)	Normoglycemia in 6 out of 9 recipients	(110)
Islets + immature DCs+ mesenchymal stem cells (MSCs)	Kidney capsule	83.33% graft survival up to 30 days	(111)
Islets + preactivated MSCs	Liver (intraportal)	5 out of 6 recipients remain normoglycemic in syngeneic model Short-lived reduction of activated NK cells in liver	(112)
Islets + MSCs	Liver (Intraportal)	Rejection free survival days of 60, 93, 105, 180 and 180 for the 5 animals tested Required 30-day reduced immunosuppression with thymoglobulin, Tacrolimus and rapamycin Downregulation of memory T cells, reduced proliferation of donor T cells and superior metabolic control observed among recipient-derived MSCs group	(113)
Islets + Intravenous injection of irradiated apoptotic donor splenocytes	Kidney capsule	Median survival time of 56.9 ± 1.8 days vs. 7.6 ± 0.8 days in control mice Tolerance attributed to generation and expansion of Tol-DCs and Tregs Tolerogenic effects independent of number of apoptotic cells infused and enhanced by administration 7 days prior to transplant	(114, 115)
Islets + infusion of ECDI-treated donor apoptotic leukocytes	Liver (intraportal)	Mean survival time for treated monkeys was extended to 85.5 days with the requirement of maintenance immunosuppression for up to 30 days >1 year tolerance for allografts in 5 out of 5 macaques associated with depletion of donor specific T and B cells and upregulation of Tr1 cells	(116, 117)

Similar to ligand conjugation on biomaterials, efforts in islet engineering have largely focused on the presentation of FasL or PD-L1 on the surface of islet cells. In this case, chemical or genetic modification of islet cells are used to modify the cell surface to resist attack from Teff cells. Yolcu et al. biotinylated islets followed by an efficient reaction with SA-FasL that remained on the surface of islets for over 1 week *in vitro* (107). Authors argued that chemical modification of islets would be preferable as transfection of cells poses concerns in terms of safety and clinical translation. Despite demonstrating indefinite survival in a syngeneic islet transplantation model, SA-FasL engineered islets achieved only 18% normoglycemia in mice over a 100-day observation period when used in an allogeneic transplantation model (107). As was the case with FasL decorated biomaterials, a short course of rapamycin was required to improve graft survival. Given previous research indicating that FasL potential role as a chemoattractant (118), which could accelerate islet damage, the authors investigated the chemotactic activity of SA-FasL engineered islets. Contrary to expectations, they observed slightly lower levels of neutrophils among graft infiltrating cells in SA-FasL engineered islets compared to SA-engineered islets (107). Similarly, islets that were chemically modified to display PD-L1 on their surface and transplanted into murine diabetic recipients on a 15 day course of rapamycin achieved long-term survival for 90% of islet recipients (108). Graft survival was associated with an increase in regulatory factors TGF-β and IL-10, and a decrease of proinflammatory cytokines in the graft microenvironment. Intragraft Tregs were critical for protection against the alloimmune response as depletion of Tregs led to prompt rejection of transplanted islets (108). On the other hand, Ma and colleagues argued that presentation of ligands such as PD-L1 are better accomplished via gene editing of cell lines since chemical modifications may be time-consuming and challenging in clinical settings given the difficulty of long-term maintenance of human islets (106). As such, authors engineered mesenchymal stromal cells (eMSCs) to express PD-L1 and CTLA4-Ig which have been shown previously to downregulate T cell activation in a nonredundant way (119, 120). Mesenchymal stromal cells were chosen as they exist in multiple tissues (121) and have been tested in numerous clinical applications (122). In a syngeneic mouse transplantation model, eMSCs co-transplanted with islets restored normoglycemia for 80% of recipients, and in the allogeneic model, the median survival time of 40 days for the eMSCs was significantly longer than the control (14 days) and non-engineered MSCs (14 days) groups (106). Without the use of any systemic immunosuppressive drugs, co-transplanted eMSCs resulted in a significantly fewer CD3+ T cells, more CD8+ T cells expressing exhaustion markers (i.e., PD-1), and more CD4+ Tregs in the graft site (123). While these results are highly encouraging for clinical translation, long-term graft survival was variable among recipients and the lack of persistence of eMSCs *in vivo* could be a potential explanation. To enhance the survival of eMSCs at the graft site, future efforts should focus on improving their access to oxygen and nutrients. Furthermore, Li et al. induced the formation of neo-islets in a diabetic non-obese diabetic (NOD) mouse model via viral transduction of the islet-defining factor neurogenin3, the islet growth factor betacellulin and PD-L1 which led to increased apoptosis of infiltrating CD4+ T cells. The combination therapy with all the aforementioned genes led to diabetes reversal for at least 14 weeks and islets with no detectable changes in their production of all islet hormones (99). Surprisingly, protection of islets in this spontaneous diabetes onset model did not rely on increased levels of Tregs, but rather downregulation of pro-inflammatory cytokines, such as IFN-γ and TNF-α (99). Although this study does not utilize a transplantation model, the finding that islet protection does not depend on Tregs suggests that the mechanism of action of PD-L1 merits further investigation.

The non-reliance on Tregs for islet tolerance stands out as an exception among the numerous studies discussed in this review. Indeed, other co-transplantation approaches include the formation of co-aggregates of islets with immunomodulatory cells such as Tregs and Sertoli cells. Iwata and colleagues used the simple and low-cost hanging drop method to produce spheroid cell clusters that can prevent allorejection without systemic immunosuppressants. Their first attempt of co-aggregates with islets involved Sertoli cells, which are large columnar cells found in the testes capable of secreting anti-inflammatory cytokines such as TGF-β (124) and killing of Teff cells via FasL expression (125). As Sertoli cells formed a barrier around islet cells, streptozotocin-induced diabetic mice received 800 co-aggregates containing 1.2 million Sertoli cells and 400 islets (109). Islets were digested into single cells to reduce the size of co-aggregates and avoid blood flow obstruction in the portal vein. Six out of seven recipients remained normoglycemic up to 100 days post the procedure (109). Histological analyses revealed significantly more insulin-positive cells in the liver and normal blood glucose regulation via metabolic testing (109). This work, while demonstrating islet protection, lacks a comprehensive investigation into the underlying mechanisms. The authors propose that the previously described functions of Sertoli cells likely play a crucial role in ensuring long-term graft survival. Given that Tregs have played an essential role in tolerance induction for islet transplantation, the next efforts of Iwata and colleagues focused on the formation of co-aggregates of Tregs and islet cells. The primary benefit of co-transplanting Tregs to create an immune-privileged niche, rather than relying on systemic infusions, lies in the fact that Tregs are relatively scarce, comprising only 2%–3% of the total circulating lymphocyte population (126). Using Tregs as a cellular therapeutic in the clinic necessitates ex vivo culture and expansion of a patient's own cells, which introduces challenges including Treg instability and their ability to transdifferentiate into Teff cells in inflammatory environments as well as the high-quality standard requirements for cellular therapies (126). The formation of co-aggregates with Tregs led to a 27% reduction in insulin production from islets suggesting that only 73% of islets were incorporated into the co-aggregates. Nonetheless, six out of nine allogeneic transplant recipients remained normoglycemic up to 120 days following the procedure (110).

Co-transplantation efforts have also explored the use of immature dendritic cells (DCs) with a tolerogenic phenotype to prevent alloimmune rejection, recognizing the critical role of costimulation in naïve T cell activation (127). As such, Long et al. co-transplanted rat islets into Balb/c mice with immature DCs and mesenchymal stem cells (MSCs) given their ability to maintain DCs in an immature state (128). As a result, 83.3% of mice receiving islets, immature DCs and MSCs survived 30 days post-transplant and had significantly better glycemic control than mice transplanted with islets alone or with either immature DCs or MSCs (111). Future work should aim to extend graft survival and investigate whether immature DCs and MSCs work cooperatively or independently toward the protection of the allograft. Another study leveraging MSCs for co-transplantation with islets, aimed to exploit their two essential properties: suppress NK cells (129) and become activated by proinflammatory cytokines such as IFN-γ, TNF-α and IL-1β (112). Hence, Ohdan and colleagues hypothesized that by co-transplanting islets with preactivated MSCs in the portal vein, the release of pro-inflammatory cytokines during IBMIR could maintain MSCs in an activated state (112) and lead to improved graft survival (112). Using a syngeneic transplantation model, authors showed that co-transplantation with preactivated MSCs significantly decreased activation markers of NK cells in the liver 3 days after the procedure (112). While the regulating effect of MSCs is not long-lasting, this may be sufficient to prevent the rapid and significant damage to islets caused during IBMIR in the first hours after the transplant. Unlike mice transplanted with islets alone or co-transplanted with naive MSCs, five out of six mice co-transplanted with preactivated MSCs achieve normoglycemia up to 45 days post the syngeneic islet transplant (112). In a recent study utilizing MSCs, Kenyon and colleagues investigated the impact of MSC source and dosage timing on graft outcomes in a nonhuman primate model of allogeneic islet transplantation (113). Researchers found that co-transplantation of recipient-derived MSCs were superior to donor or third-party MSCs in prolonging rejection-free days and overall islet survival. The optimal treatment regimen involved intrahepatic co-transplantation of islets with 1 × 10^6^ MSCs/kg on day 0, followed by intravenous infusions of 2 × 10^6^ MSCs/kg on days 5, 11, 18, and 28 post-transplant, combined with a 30-day reduced immunosuppression protocol (113). Long-term graft survival was associated with significant downregulation of memory T cells, decreased anti-donor T cell proliferation in mixed lymphocyte reactions, and a trend toward increased regulatory T cell to conventional T cell ratios (113). However, the authors noted limitations, including the need for further studies to elucidate the impact of anti-inflammatory agents on MSC efficacy [since MSCs are activated by inflammatory cytokines (130)], the potential benefits of incorporating costimulatory blockade to enhance graft survival (113), and the challenge of translating a protocol requiring multiple MSC infusions to clinical practice.

Finally, donor apoptotic cells have also been investigated as a promising tool for inducing transplantation tolerance due to their unique immunomodulatory properties. Previous studies have explored the clearance of apoptotic cells and its role in maintaining peripheral tolerance to self-antigens, as well as the potential to provide donor antigens to recipient's APCs and induce antigen- specific tolerance (131). Unlike necrotic cells, which trigger inflammation, apoptotic cells are cleared silently by phagocytes and create an immunosuppressive environment. This is mediated through the release of anti-inflammatory cytokines like TGF-β and IL-10, the downregulation of NF-κB as well as the expression of “eat-me” signals like phosphatidylserine exposure that promote their efficient clearance (132). Hence, apoptotic cells can be utilized to deliver donor antigens in a tolerogenic manner. As such, Wu et al. showed that intravenous administration of 10^7^ apoptotic donor splenocytes, induced by ultraviolet-B irradiation and confirmed by annexin V and propidium iodide staining, one week prior to islet transplantation significantly prolonged allograft survival in streptozotocin-induced diabetic BALB/c mice receiving C57BL/6 islets (56.9 ± 1.8 days vs. 7.6 ± 0.8 days in controls) (114). Infusion with live splenocytes 1 week prior to islet transplantation did not prolong graft survival. Long-term islet survival was associated with the induction of tolerogenic dendritic cells (Tol-DCs) and expansion of Tregs in diabetic recipients (114). The authors demonstrated a critical reciprocal interaction between Tol-DCs and Tregs, with Tol-DCs promoting Treg expansion via PD-L1, and Tregs maintaining the tolerogenic state of DCs through IL-10 and TGF-β (114). Depletion of either DCs or Tregs abrogated the tolerance-inducing effects of apoptotic cell infusion (114). In another work by Mougel and colleagues, it was demonstrated that infusing 5 × 10^6^ or 50 × 10^6^ apoptotic splenocytes into diabetic mice recipients 7 days prior to transplantation resulted in similar delays in islet rejection, indicating that increasing the number of apoptotic cells did not enhance the tolerogenic effect (115). Additionally, they found that administering the apoptotic cells 7 days before transplantation was more effective in prolonging graft survival compared to infusion on the day of transplantation (115). These results suggest that donor antigens, rather than the apoptotic cells themselves, are the critical component for tolerance induction. The timing of injection 7 days prior to transplantation likely allows for processing by splenic APCs and induction of regulatory immune populations (131).

Apoptotic cell infusion has also shown promise in nonhuman primate studies. Unlike in murine studies where pre-infusion of apoptotic cells 7 days prior to transplantation resulted in prolonged graft survival, Lei et al. showed that portal vein infusion of ethylene carbodiimide (ECDI) treated donor lymphoid cells (4.5 × 10^8^–3.75 × 10^9^ cells) in monkeys on the day of transplantation resulted in mean survival time of 85.5 days compared to 13.5 days for the control group (116). While apoptotic donor lymphoid cells generated and expanded CD4+ CD25+ Foxp3+ cells, maintenance immunosuppression with rapamycin and anti-IL6R was required for up to 30 days (116). On the other hand, Singh et al. opted for two infusions of donor apoptotic leukocytes 7 days prior to transplantation and 1 day after, showing >1 year tolerance to islet allografts in 5 of 5 rhesus macaques under a short immunotherapy regimen (117). Long-term graft survival was attributed to depletion of donor-specific T and B cell clones and potent, sustained regulation involving antigen-specific Type 1 regulatory (Tr1) cells (117). As these two studies demonstrate the overall safety of donor apoptotic leukocyte infusion in large animal models and provide the foundation for clinical translation, risks related to prior sensitization in diabetic recipients should be carefully evaluated (131).

Islet engineering and co-transplantation strategies demonstrate that islets can be protected without the need for physical barriers between the host and the islets or systemic immunosuppression. Nonetheless, translation may be limited by factors such as the large number of immunomodulatory cells required for large animal models and humans, risks of sensitized recipients with donor cells and the long-term maintenance of cellular function in the graft microenvironment (123).

## 5 Conclusion, challenges, and outlook

While the FDA has recently approved Lantidra, the first allogeneic pancreatic islet cell therapy aiming to restore physiological glucose control (133), many barriers remain for islet transplantation to be the standard of care for all T1D patients. Two of the most pressing challenges to widespread adoption of islet transplantation in clinical settings are the scarcity of donor islet cells and the necessity for systemic immunosuppression to prevent rejection. Furthermore, insurance coverage for islet transplantation is necessary to make it an affordable option for every patient (134). In addition to the high cost, another hindrance to islet transplantation in the United States specifically is the current FDA classification of allogenic islets as a biologic drug which requires extensive and costly Biologics License Application (BLA) processes (135). These stringent regulatory requirements have dramatically reduced islet transplantation procedures from 179 between 1999 and 2005 to just 11 patients between 2016 and 2019 (135). In contrast, other countries recognize islets as minimally manipulated tissue and have made islet transplantation a standard clinical procedure (136). Witkowski et al. argue that the current US regulatory framework is scientifically obsolete and propose updating regulations to regulate islets under Part 1271, allowing oversight by the Organ Procurement and Transplantation Network (OPTN)/United Network for Organ Sharing (UNOS), which would make the procedure more accessible, affordable, and potentially life-changing for patients with type 1 diabetes suffering from severe hypoglycemic episodes (135).

To address the scarcity of donor islet cells, efforts to produce islet cells from stem cells have exploded in the past two decades (55, 137), leading to more control over β-cell source and an opportunity to reduce allergenicity by tailoring the donor MHC to the transplant recipient (138). Furthermore, advances in gene editing using CRISPR-Cas9 technology can enable the generation of porcine islets and hypoimmune stem cell derived beta-like cells (139, 140) with diverse genetic modifications, including reduced immunogenicity and enhanced insulin production while providing a constant supply of islet donors (4). Given that CRISPR-Cas9 technology was recently employed in the first FDA-approved gene therapy for sickle cell disease (141), there is justified optimism about its potential to address the islet donor shortage in the near future. However, while the breakthrough is promising, careful consideration is required as gene-editing advancements move toward the clinic, especially with the use of viral vectors which could trigger immune reactions.

**Figure 2:**
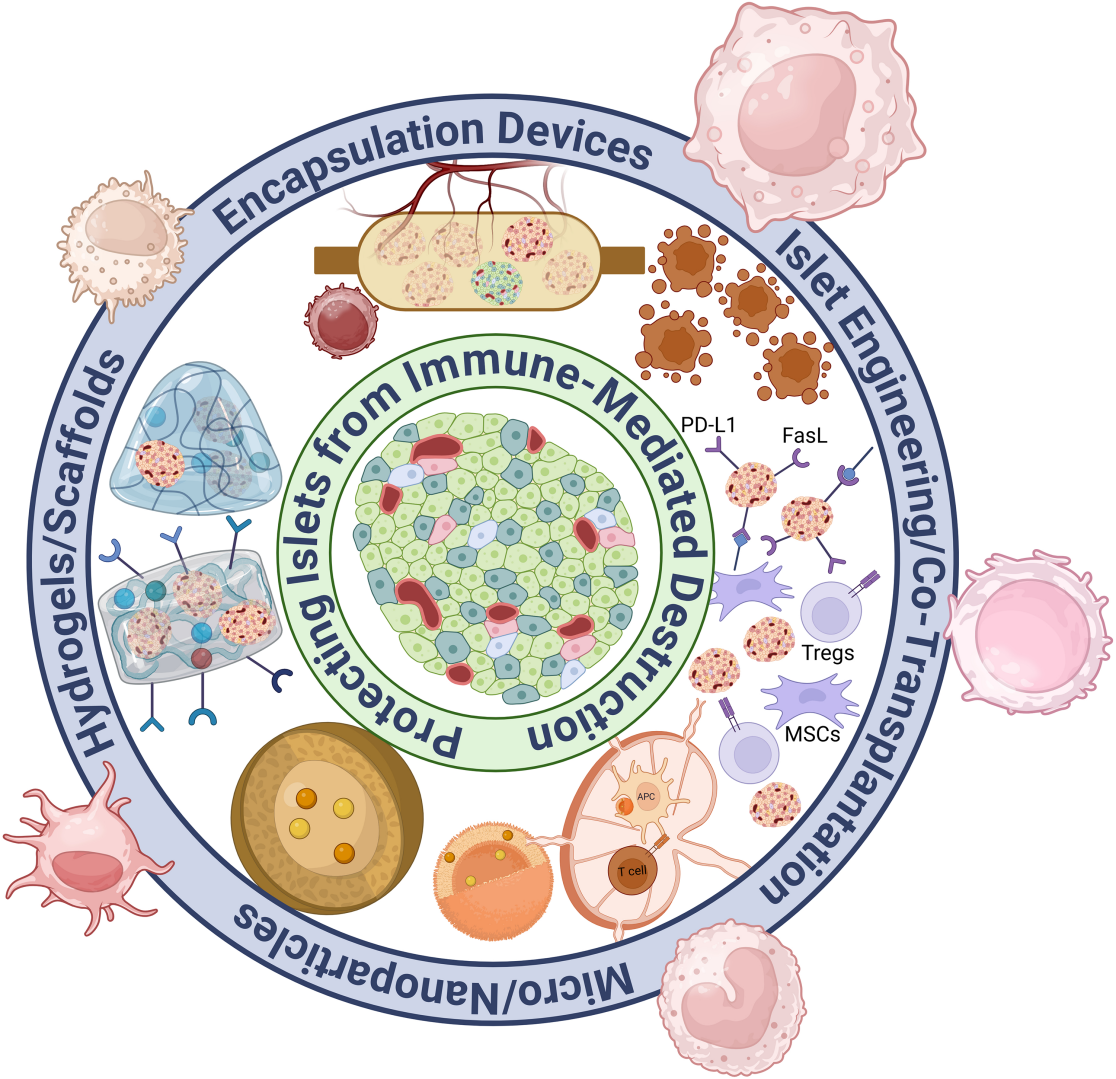
Overview of immunomodulatory approaches for islet transplantation.

With these groundbreaking innovations to expand islet availability, the focus of research for islet transplantation has shifted toward eliminating the need for systemic immunosuppression, which remains a major barrier to widespread clinical adoption. Achieving antigen-specific tolerance for transplanted islets has become the primary goal, as this would allow for long-term graft survival without the severe side effects linked to systemic immunosuppressive therapies. Systemic immunosuppression risks exposing the patient to opportunistic infections and malignancies as well as causing islet graft failure due to drug toxicity (8). This review briefly discussed islet encapsulation devices, with a major focus on biomaterial approaches, islet engineering, and co-transplantation strategies. These innovative immunotherapies aim to overcome rejection, prolong graft survival, and improve the quality of life for islet transplantation recipients, potentially freeing them from lifelong immunosuppressive therapy and its negative impacts on their lifestyle. These recent research advances for islet transplant immunotherapy are summarized in Figure 2.

Cell encapsulation devices such as VC-02 and Vertex's VX-264 attempt to find the right balance between immunoisolation and long-term integration with the host. While trials are still ongoing for VX-264, there are certainly concerns regarding the foreign body reaction which can lead to reduced cell viability due to the formation of a dense fibrous capsule acting as a barrier to the diffusion of oxygen and nutrients (142, 143). Furthermore, given the presence of channels on these encapsulation devices allowing diffusion of small molecule drugs, combining systemic immunosuppression and stem cell-derived sources will raise serious safety concerns regarding the possible formation of teratomas (144, 145). As these encapsulation devices advance toward clinical use, careful attention must be paid to selecting a transplant site that allows for ease of retrieval in case of tumor formation. This consideration is vital to ensure both patient safety and the ability to quickly access and remove the devices if complications arise, minimizing the risk of infection or other adverse events. Several promising transplant sites are under active investigation, including the subcutaneous space, the omentum, the anterior eye chamber and the intramuscular space. These extrahepatic sites are reviewed in greater detail here (146). The subcutaneous space is relatively avascular, and as such its use as a transplant site has mainly required the use of extracellular matrix components (90) to promote vascularization and full engraftment of the islets. Given the abundant presence of innate and adaptive immune cells in the subcutaneous microenvironment, systemic or localized immunosuppression would likely be necessary to prevent rejection of transplanted islets (146). Unlike the subcutaneous space, the omentum offers the advantage of being highly vascularized and potentially immune privileged; however, the potential requirement for a laparotomy for islet transplantation raises concerns about post-surgical complications such as wound infections and impaired healing (147). The anterior chamber of the eye is another extrahepatic site that has been gaining significant research interest due to its unique characteristics. This site boasts a high vascularization density and possesses an immune privileged status attributed to its aqueous humor which inhibits T-cell proliferation (148) and IFN-γ production (149). Despite potential risks such as blindness, cataract formation (150) and collagen deposition (151), two ongoing clinical trials are evaluating the safety and efficacy of islet transplantation in the eye for both visually impaired diabetic and healthy patients (146). Finally, the intramuscular transplantation site also offers several advantages for islet transplantation. It reduces IBMIR-related islet loss, allows multiple transplant locations and provides easy access for repeated implantation and retrieval of the graft (146). Despite its advantages, the intramuscular transplantation site shares limitations with the subcutaneous space, notably a hypoxic and immunogenic microenvironment (146). All in all, the optimal site for islet transplant remains a subject of debate. Improving islet graft survival will likely require a highly vascularized, oxygen-rich and immune regulated environment, potentially achieved using biomaterials or other encapsulation devices that elicit minimal foreign body reactions.

In other research endeavors, surface-modified biomaterials or releasing immunomodulatory compounds provide multifaceted ways to prolong islet graft survival by either locally protecting the islet cells from the host's immune system or modulating the immune response from the draining lymph nodes. The latter structures are of particular interest as APCs presenting donor antigen from the site of transplantation migrate to lymph nodes where they prime T cells, which then migrate to the graft to mediate rejection (152). Biomaterials offer multiple advantages given their ability to mimic functions of recipient's regulatory cells via the delivery of immunomodulatory agents or the presentation of tolerogenic ligands and their readiness as an “off-the-shelf” product. However, questions remain regarding the sustainability of biomaterial approaches given the possible shedding of FasL or PD-L1 ligands from the biomaterial's surface and the dwindling levels of loaded drugs and proteins overtime. Additionally, long-term survival of the graft using loaded or surface-modified hydrogels or scaffolds required a short course of immunosuppression which needed to be extended in large animal models (76). This requirement for systemic immunosuppressants emphasizes the need for biomaterial approaches to go beyond the islet microenvironment and possibly target other immune players present in the local draining lymph nodes. As such, there have been attempts to formulate drug/antigen loaded microparticles and nanoparticles capable of accumulating in the lymph nodes in order to downregulate CD4+ T cell activation (56) or upregulate donor-specific Tregs (66). Donor or antigen-specific Tregs are usually considered superior to polyclonal Tregs for transplantation due to effective homing and activation in the lymph nodes (153). Although these approaches might need to be combined with local immunomodulatory biomaterial strategies, their application in large animal models remains uncertain. The significantly greater number of lymph nodes in larger animals compared to mice could make it more challenging to precisely target the appropriate lymph nodes for desired immunomodulation. This increased complexity, combined with the potential necessity for ultrasound guidance to safely perform intranodal injections in clinical settings (154), present challenges that must be addressed for successful translation to islet transplant recipients. Since these biomaterial approaches aim to establish localized immune tolerance without compromising functional protective immunity, it is essential to prioritize more robust preclinical *in vivo* models, such as skin graft tests or the transplantation of donor and third-party islets in different physiological locations to better appreciate the systemic or localized nature of the induced tolerogenic effects (155). These models are preferable to mixed lymphocyte reaction *in vitro* tests, which are typically limited to splenocytes and may not fully capture the complexity of immune response. Additionally, there have been clear challenges translating the success of transplant immunotherapies from mice to larger animal models and humans. The chemically induced diabetes mouse models offer more consistent diabetes induction and enhanced control over experimental conditions; however, they lack the complex autoimmune responses observed in spontaneous T1D onset, limiting the ability to test newly formulated therapies targeting autoreactive memory T cells. Nonetheless, there have been recent efforts focused on targeting memory T cells in recurrence autoimmunity, with strategies such as blocking IL-7 and IL-15 signaling showing promise in reversing new-onset autoimmune diabetes in NOD mice (156). These approaches aim to modulate the autoreactive memory T cell compartment, which is critical for maintaining long-term remission and potentially restoring immunologic tolerance in T1D. On the other hand, while the NOD mouse model provides a valuable model for studying the autoimmune aspects of type 1 diabetes (157), its complex immune responses, high variability, and resistance to islet engraftment make it less suitable for islet transplantation research. Therefore, the shift towards humanized mouse models is crucial, as murine islets consistently demonstrate superior outcomes compared to human islets (158), highlighting the need for more accurate representations of human islet biology in diabetes research. Finally, efforts aiming to co-load antigen and immunomodulatory agents for islet transplantation or T1D prevention (66) face the obvious hurdle that the initial immune events leading to the destruction of islet cells in T1D still remain unknown (159). Hence, the development of delivery systems releasing antigens and active molecules for the induction of antigen-specific tolerance will demand more research focus on the loss of self-tolerance via autoantigen recognition followed by T cell mediated destruction of the β-cell population. On the other hand, tolerogenic strategies that do not require prior knowledges of autoantigens for T1D present an advantage for clinical translation (71).

Islet engineering and co-transplantation with immune regulating cells also represent a promising approach to create an immune privileged environment for the transplanted islets. Chemically modifying islets to display ligands like FasL and PD-L1 (107, 108) on their surface allows the islets to induce death of activated T cells or upregulation of Tregs, eliminating the need for third-party biomaterials or accessory cells for immune defense. Like biomaterial strategies, chemically modified islets could be developed as an “off-the-shelf” product, utilizing stem-cell-derived sources for the modified islets. However, genetic modification of islets or immunomodulatory cells can be cumbersome and fairly difficult to translate to the clinic given safety concerns surrounding the continuous expression of potent regulatory ligands (107). Additionally, questions regarding the stability of genomic and phenotypic changes demand further investigation. Unlike cargo-releasing biomaterials, which may eventually deplete their payload, co-transplantation of immunomodulatory cells offers a continuous source of cytokines and ligand expression, providing sustained protection for the transplanted islets. While mesenchymal stem/stromal cells are plentiful in many tissues (121), Tregs and Sertoli cells are comparatively rare (126, 160), despite the need for a substantial number of cells per transplanted islet to achieve therapeutic efficacy. Additionally, these co-transplanted cells must be harvested from the transplant recipient, expanded *ex vivo*, and maintained under strict good manufacturing practices until the transplantation procedure. Hence, more efforts should be directed towards ensuring the proper acquisition and maintenance of these immunomodulatory cells, in addition to assessing potency for improved safety and efficacy. Regarding the use of apoptotic cells for allogeneic islet tolerance, it is noteworthy that donor specificity does not appear to be essential, as the delivery of apoptotic cells from any origin can induce tolerance to the antigens presented by these cells to recipient APCs (131). As the timing of infusion was shown to be critical for optimal performance, the number of infused apoptotic cells seem to have little effect on long-term graft survival unlike MSCs and Tregs (115). Additionally, unlike MSCs or Tregs which must stable for long periods of time to preserve the tolerogenic milieu of the islets, apoptotic cells may not need to remain in circulation for extended periods of time in circulation as their phagocytosis is key to the subsequent expansion of tolerogenic DCs and Tregs. Nonetheless, there are still several obstacles to clinical translation of apoptotic cells infusions for islet transplantation. These include the risk of prior sensitization, the need to carefully control apoptosis induction, and the limited practicality of using donor apoptotic cells from donors with underlying medical conditions (131).

In summary, the achievement of antigen-specific tolerance for long-term graft survival of allogeneic islet cells will certainly require a transdisciplinary approach combining a drug-releasing or surface-modified biomaterial with islets expressing potent regulatory ligands and co-transplanted with readily available accessory cells. Future research must place a greater emphasis on identifying all possible autoantigens involved in T1D pathogenesis to ensure that the engineered solutions for allogeneic islet transplantation comprehensively address the complexities of this multifaceted disease.
